# In and out among the Hospitals

**Published:** 1888-09-22

**Authors:** 


### In and Out Among the Hospitals.

By a Secretary of Ten Years' Standing.

Copies of Annual Reports and Rules, Accounts of Meetings, Changes in the Staff, &c., should be addressed The Editor, The Lodge, Porchester Square, W.

### HALIFAX INFIRMARY.

This is the principal institution for the relief of the sick in a very large manufacturing district, and not only are patients treated in the wards, but a very large number are seen as out-patients, and also at their homes in the vicinity of the hospital. Five thousand six hundred and forty-one visits were paid to this latter class of patients during the last twelve months, and it may be assured that there is no medical relief which is so acceptable to the poor as this system of seeing them in their own homes. It may be doubted if it is to their advantage in all instances, but the reluctance to go into hospital, less though it is now than formerly, still is very strong among a large number of those who need medical aid and are unable to provide it for themselves. The Halifax Infirmary is one of those that has not made much progress in providing improved accommodation for the nurses in its employ, but it is only fair to state that the management is fully aware of the necessity that exists for an improvement in this department, and it is only a matter of expense that something has not been done ere this. At present some of the nurses are put up in a private house in the neighbourhood of the hospital, which is a system much to be deprecated, and others are provided with dormitories that are far from meeting the requirements that are now considered necessary in every well-regulated establishment. The expenditure, after deducting cost of out-patients, &c., was £4,663; and as the daily average number of patients was 82, this gives the cost per bed occupied at £56 17*s.* 6*d.* The total receipts were £5,143, and include £1,777 from the workpeople in the various mills and workshops in Halifax. The number of in-patients was 960, and of out-patients 5,323.

### GLASGOW ROYAL INFIRMARY.

This is one of the largest general hospitals in Great Britain, and the daily average number of patients resident last year was 493. It appears that patients are retained much longer in the wards than is usual in similar institutions in England, the average residence being over 36 days, but it must be taken into account that, as a rule, only very urgent cases are admitted, all others being treated in the out-patient department. Scotchmen look closely into the merits of a case before granting the benefits of their charity, and it will be found that there is less indiscriminate relief afforded than we are used to see. The Glasgow Royal enjoys, probably, a larger annual subscription list than any other infirmary. Last year it amounted to £12,241 9*s.* 3*d.*, and the total income to £39,903. This includes £20,732 from legacies, and it is instructive to observe that in the last twelve months no less than 38 legacies were bequeathed to this hospital. The residents of Glasgow, in their way, show their appreciation of the benefits that the Infirmary bestows upon the town. The expenditure was £25,018. Deducting extraordinary expenses and 1*s.* for each out-patient, the cost per bed occupied is £43 10*s.* 4*d.* The Provost of Glasgow is always at the head of the board of managers, and an active interest is taken in the charity by all the principal residents and by the workmen, who, through their foremen, contribute largely to the funds. A large medical school is attached to the hospital, and a great effort is being made to introduce into the proposed "Scottish Universities Bill" a clause converting the medical school of the Infirmary into a College of Glasgow University. Lord Lothian, Secretary for Scotland, is interesting himself in the subject, the importance of which on behalf of the students cannot be over-estimated. The new nurses' home is now complete, and it is expected that in the course of the present year all will be in working order. There were 4,790 in-patients and 34,060 out-patients treated during the last year. The report is excellently compiled, and will be found clear and intelligent to every subscriber.

### LEICESTER INFIRMARY.

The record of this hospital is one of steady advance, increase in income, increase in number of patients, and improvement in the structure and administration. Two most interesting and valuable presentations have been lately made to the infirmary. The widow and friends of the late Dr. Buck, who for many years was one of the physicians, have built a handsome memorial chapel in the grounds on the western side. It is a plain brick building in the Early English style, consisting of a nave, apse, and organ-chamber, and will seat one hundred persons. The total cost, including a new organ, was £1,100; and a commodious porch has been erected on the east side of the building by the friends of the late Dr. Pearce, which has greatly improved this side of the hospital. The extension of the west wing has been completed, whereby very much better accommodation has been provided for the nurses. Not only in London, but also in the provinces is this improvement in the treatment of nurses being effected, and before long it may be anticipated that there will not be found a hospital in the country that does not supply separate sleeping apartments for each nurse. The daily average number of in-patients was 172½, and the expenditure, after deducting the cost of out-patients and extraordinary expenditure, £8,524, which gives the cost per bed occupied £49 11*s.* 2*d.* The entire receipts were £20,436, which includes £11,115 from legacies. This happy state of affairs has enabled the governors not only to clear off all debts upon the building, but to invest the very handsome sum of £7,745. There were 2,790 in-patients and 11,453 out-patients, which is the highest number upon record at this institution. It is gratifying to see that large support is given by the friendly societies and the working classes generally.

### PAISLEY INFIRMARY.

This hospital has been doing good work during the last twelve months; for some years its managers have been hampered with an outstanding debt, which has been a source of great anxiety; but an effort has at last been made to put matters right, and last year a special fund was raised to pay it off. This, together with very great economy in the administration, has enabled the governors to almost extinguish the debt. There is now only a sum of £356 owing, and it is hoped that this will disappear before another report is published. It should be pointed out that two most munificent annual subscriptions are received at this hospital; Messrs. Coats and Co. subscribe £150, and Messrs. Clark and Co. £100. The Fever Hospital in connexion with the Infirmary has been unusually full, and it is sad to record that several of the nurses contracted fever, one of whom succumbed to the disease. Dr. Taylor resigned his appointment, after many years' services as an active member of the staff, and has been elected to the consulting staff. The total receipts are £3,277 9*s.* 9*d.*, and the expenditure £3,000 4*s.* Deducting 1*s.* per each out-patient, the cost per bed occupied is £28 1*s.* 7*d.* The daily average number of patients is 97, while 1,110 in-patients and 5,526 out-patients were treated.

